# Ectopical expression of bacterial collagen-like protein supports its role as adhesin in host–parasite coevolution

**DOI:** 10.1098/rsos.231441

**Published:** 2024-04-03

**Authors:** Benjamin Huessy, Dirk Bumann, Dieter Ebert

**Affiliations:** ^1^ Department of Environmental Sciences, Zoology, University of Basel, Basel 4051, Switzerland; ^2^ University of Basel, Basel 4056, Switzerland

**Keywords:** *Daphnia magna*, *Pasteuria ramosa*, *Bacillus thuringiensis*, collagen-like proteins, host–parasite interactions, fusion protein

## Abstract

For a profound understanding of antagonistic coevolution, it is necessary to identify the coevolving genes. The bacterium *Pasteuria* and its host, the microcrustacean *Daphnia*, are a well-characterized paradigm for co-evolution, but the underlying genes remain largely unknown. A genome-wide association study suggested a *Pasteuria* collagen-like protein 7 (Pcl7) as a candidate mediating parasite attachment and driving its coevolution with the host. Since *Pasteuria ramosa* cannot currently be genetically manipulated, we used *Bacillus thuringiensis* to express a fusion protein of a Pcl7 carboxy-terminus from *P. ramosa* and the amino-terminal domain of a *B. thuringiensis* collagen-like protein (CLP). Mutant *B. thuringiensis* (Pcl7-*Bt*) spores but not wild-type *B. thuringiensis* (WT-*Bt*) spores attached to the same site of susceptible hosts as *P. ramosa*. Furthermore, Pcl7-*Bt* spores attached readily to susceptible host genotypes, but only slightly to resistant host genotypes. These findings indicated that the fusion protein was properly expressed and folded and demonstrated that indeed the C-terminus of Pcl7 mediates attachment in a host genotype-specific manner. These results provide strong evidence for the involvement of a CLP in the coevolution of *Daphnia* and *P. ramosa* and open new avenues for genetic epidemiological studies of host–parasite interactions.

## 1. Introduction

Antagonistic coevolution between hosts and parasites has been suggested to be a major driver in evolution, presumably underlying diverse biological phenomena, such as the extraordinary genetic variation at the major histocompatibility complex of jawed vertebrates and R-genes in plants, the parasite hypothesis about the evolution of sexual selection, the evolution of genetic recombination and the evolution of immune systems [1–3]. While great progress has been made in our understanding of coevolution and its consequences at the phenotypic level, much less is known about the underlying genetics [4–6]. However, current theories about the mechanism of coevolution are genetic models, such as the selective sweep model, where new beneficial mutations sweep to fixation in both antagonists, and balancing selection models, where alleles at specific loci interact in a manner that generates negative frequency-dependent selection [7,8]. Therefore, to test these models and understand the evolutionary dynamics during coevolution, we need to identify the genes involved in host–parasite interactions. This is particularly challenging in non-model systems, where genetic tools are largely lacking.

A prime model system for coevolution research is the water flea *Daphnia* and the bacterial parasite *Pasteuria ramosa. P. ramosa* is a highly virulent obligate parasite of its planktonic crustacean host. It cannot be cultured outside its host. For the *Pasteuria–Daphnia* system, coevolutionary dynamics have been demonstrated in natural and experimental settings, with negative frequency-dependent selection being the main explanation for the observed dynamics [9–11]. The system is renowned for its strong genotypic infection specificity [12–14], but the genes responsible for this specificity have not been identified; even so candidates have been suggested for both host and parasite [15–17]. During infection, dormant *P. ramosa* endospores are taken up by the filter-feeding *Daphnia* and shed their exosporium, revealing numerous peripheral fibres [13]. These activated spores attach to the cuticles of susceptible *Daphnia*, most commonly to the oesophagus or the hindgut wall [15,18,19]. The current understanding is that the peripheral fibres of the activated spores may be collagen-like proteins (CLPs) that act as adhesins on surface components of the *Daphnia* epithelium.

CLPs, proteins with high similarity to eukaryotic collagens, have been identified in a range of prokaryotes [20–22] including human–pathogenic species such as *Bacillus anthracis* [23]*, Legionella pneumophila* [24] and several *Streptococcus* species [25]. CLPs typically attach to cell walls [26,27] and contain a rod-shaped collagenous domain near the cell surface [28–30]. Research on bacterial CLPs indicates that they play a pivotal role in host–pathogen interactions during the initial stages of infection for attachment to host cells and surfaces [31–36]. While most bacteria contain only a few genes that encode for CLPs [21], the endospore-forming bacteria of the Gram-positive *Pasteuria* genus carry up to 50 CLP-encoding genes [20,37], one of them, *Pasteuria* collagen-like protein 7 (Pcl7) of *P. ramosa*, being suggested to be responsible for the highly specific interaction with the host and may play an important role in their coevolution [19]. Conducting a genome-wide-association study Andras *et al*. [19] discovered multiple phenotype-associated sequence polymorphisms in the *P. ramosa pcl7* gene encoding for a Pcl7. In its C-terminal domain (CTD), *pcl7* contains seven single-nucleotide sequence polymorphisms that correlate perfectly with infection phenotype and that encompass considerable changes in the size, hydrophobicity and charge of the respective amino acids. These findings suggest that Pcl7 may be crucial for spore–host attachment and, furthermore, that sequence variation in Pcl7 may be important for determining the high specificity of the bacterial spore’s adhesion to the host epithelium in the oesophagus. The aim of this study was to test the hypothesis that *pcl7* is responsible for attachment to the host’s oesophagus through experimental manipulation of Pcl7.

Genetic engineering on *P. ramosa* has not yet been successful because its rigid exosporium resists lysis and degradation [38], preventing us from generating *pcl7* mutants. However, CLPs can be engineered in the *Bacillus cereus* group [39–43], providing a heterologous system for studying *pcl7* and other *Pasteuria* factors [44]. BclA, a structural homologue of Pcl7 [19], is part of the exosporium in the entire *B. cereus* group including *Bacillus thuringiensis* (*Bt*), a species with well-developed techniques for laboratory experiments. Members of the *B. cereus* group develop spores encapsulated by an exosporium composed of two defined layers [29]: a primary basal layer and an outermost hair-like structure [45,46]. BclA makes up the majority of this hair-like structure [23]. It is expressed at the spore surface late during sporulation and requires the specific amino acid sequence motif ‘LVGPTLPPIPP’ for incorporation into the exosporium [47].

Here, we used the collagen part of BclA as a display platform for the CTD of Pcl7 in *B. thuringiensis* to obtain a surrogate parasite that displays the key part of Pcl7 on its surface. Analogous display systems have already been used to express functional fusion proteins [48–50]. We showed that the Pcl7 CTD mediated attachment of the surrogate parasite spores to the oesophagus wall of *D. magna* and that this part of a single protein was sufficient to recapitulate the host genotype specificity of the donor *P. ramosa* clone. These data demonstrate the key role of Pcl7 CTD in this paradigmatic host–pathogen system.

## 2. Material and methods

### 2.1. Media

*Escherichia coli* DC10B (table 1) was cultured in lysogenic broth (LB; 10 g l^−1^ bacto tryptone, 5 g l^−1^ yeast extract and 10 g l^−1^ NaCl) medium. *B. thuringiensis* was cultured in tryptic soy broth (TSB; 30 g l^−1^ bacto TSB) medium. LB-lowsalt (LB-ls; 10 g l^−1^ bacto tryptone, 5 g l^−1^ yeast extract and 5 g l^−1^ NaCl) was used to prepare electrocompetent cells. For electroporation, Super-Optimal broth with Catabolite Repression (SOC; 20 g l^−1^ tryptone, 5 g l^−1^ yeast extract, 0.5 g l^−1^ NaCl, 4.8 g l^−1^ MgSO_4_, 0.186 g l^−1^ KCl and 3.6 g l^−1^ glucose) was used. For selection, we used LB agar plates (10 g l^−1^ bacto tryptone, 5 g l^−1^ yeast extract, 10 g l^−1^ NaCl and 15 g l^−1^ agar) and TSB agar plates (30 g l^−1^ bacto TSB and 15 g l^−1^ agar) with erythromycin at a final concentration of 5 µg ml^−1^, ampicillin at a final concentration of 100 µg ml^−1^ and anhydrous tetracycline at a final concentration of 8 µg ml^−1^.

**Table 1.** Material used in the study.

clone	country of origin (collection site)	C1 resistotype
A. *Daphnia magna* genotypes (=clones) used in the study		
HU-HO-2	Hungary	susceptible
NO-AA-1	Norway	susceptible
RU-AST1-1	Russia	susceptible
CH-H-2016-h-34	Switzerland	susceptible
FI-Xinb3	Finland	resistant
DE-G1-106	Germany	resistant

strain or plasmid	characteristics	reference
B. Bacterial strains and plasmids used in this study		
bacterial strain		
*B. thuringiensis* 407 Cry^-^	acrystalliferous *B. thuringiensis* strain	[51]
*E. coli* DC10B	mcrA Δ(mrr-hsdRMS-mcrBC) φ80lacZΔM15 ΔlacX74 recA1 araD139 Δ(ara-leu)7697 galU galK rpsL endA1 nupG Δdcm	[52]
*P. ramosa C1 and C19*	cloned lines of *P. ramosa*, isolated from natural populations	[13]
plasmid		
pBKJ223	plasmid-encoded homing restriction enzyme I-SceI, promoting the second homologous recombination event in procedure for making markerless deletion mutant, Tet^r^	[53]
pMAD-I-SceI	plasmid used for making deletion mutants, containing I-SceI restriction site that can be cleaved by the homing endonuclease I-SceI, Apr, Eryr	[54]

*Notes:* Six *D. magna* clones with different resistance phenotypes from the C1 *P. ramosa* clone were maintained in an artificial culture medium (ADaM) under standard laboratory conditions (20°C, 16:8 day:night cycle, *Tetradesmus obliquus* as food) as previously described in [55].

### 2.2. Preparation of electrocompetent cells and electroporation

To prepare electrocompetent cells, 100 ml of fresh LB-ls was inoculated at OD_600 nm_ 0.01 from an overnight culture and grown to OD_600nm_ 0.08 at 37°C, 180 r.p.m. The culture was distributed to two 50 ml falcon tubes and put on ice for 20 min. The tubes were spun down at 4°C, 8000 r.p.m. for 8 min, and the supernatant was removed. The pellets were then washed with 30 ml of cold sterile water containing 15% glycerol (AppliChem, A1123,1000), and the tubes were spun down at 4°C, 8000 r.p.m. for 8 min. This washing step was repeated twice. The supernatant was discarded, and the pellet was suspended in 1 ml of cold sterile water containing 15% glycerol. Finally, 100 µl aliquots were stored at −80°C or used directly for electroporation.

For electroporation of plasmid DNA, DNA (100 ng) was added to thawed, electrocompetent cells (100 µl) in a 1 mm electroporation cuvette on ice. For *E. coli* DC10B, a single pulse at 1.8 V (Gene Pulser Xcell Electroporation Systems, Bio-Rad Laboratories) was applied. For *B. thuringiensis,* a single pulse at 2.5 V was applied. Immediately after the pulse, 900 µl of pre-warmed SOC media was added, and the entire volume was transferred to a fresh 1.5 ml Eppendorf tube. The tube was incubated and shaken at 37°C, 180 r.p.m. for 1 h. Samples were spun down for 4 min at 11 000 r.p.m. We removed 900 µl of supernatant and plated the remaining volume (100 µl) on LB agar containing the respective antibiotics. Plates were incubated overnight at 37°C.

### 2.3. Isolation of genomic DNA

Genomic DNA was isolated using a DNeasy Blood & Tissue Kit (Qiagen GmbH, Hilden, Germany). A 2 ml overnight culture of *B. thuringiensis* in TSB media was spun down at 8000 r.p.m. for 4 min. The supernatant was discarded, and the pellet was resuspended in 200 µl of AL Buffer and 20 µl of proteinase K. The sample was incubated at 56°C, 500 r.p.m. for 10 min and 200 µl of >99% ethanol was added to it. It was then mixed thoroughly, transferred to a DNeasy Mini spin column and spun down at 10 000 r.p.m. for 1 min; the flow through was discarded. Then 500 µl of Buffer AW1 was added and spun down at 10 000 r.p.m. for 1 min, and the flow through was discarded. Finally, 500 µl of Buffer AW2 was added and spun down at 13 200 r.p.m. for 1 min. The column was transferred to a fresh 1.5 ml Eppendorf tube, and the DNA was eluted with 50 µl sterile water, incubated at room temperature for 1 min and centrifuged at 10 000 r.p.m. for 30 s. The concentration of DNA was measured using a Colibri Microvolume Spectrometer (Berthold Technologies, Bad Wildbad, Germany).

### 2.4. Molecular biology

Plasmids were constructed by Gibson Assembly [56]. Flanking regions (~750 bp) of the target locus were PCR-amplified from genomic DNA (with primers 1 and 2 as well as 5 and 6, table 2) using Phanta Max Super-Fidelity DNA Polymerase (Vazyme Biotech, Nanjing, China), and primers were designed with SnapGene (v. 5.2, Gibson assembly tool). The 501 bp C-terminal *pcl7* sequence was synthesized (LubioScience GmbH, Zurich, Switzerland), and PCR-amplified using primers 3 and 4. The pMAD-I-SceI vector was amplified with primers 7 and 8 in a long-range PCR. The resulting three fragments and the vector were fused using the Hifi DNA Assembly Master Mix (New England Biolabs, Ipswich, MA) at 50°C for 1 h. The reaction mix contained approximately 40 ng of each fragment and 150 ng of vector for a total volume of 20 µl. *E. coli* DC10B was transformed with the resulting product. Plasmid DNA was purified from an overnight culture using a plasmid miniprep kit (ZymoPURE, ZymoResearch). Sequence-verified plasmids were electroporated into *B. thuringiensis*. Cells were incubated at 28°C for 1 h and plated on TSB agar containing 5 µg ml^−1^ erythromycin.

**Table 2.** Primers used in this study.

	primer name	sequence 5′ → 3′
1	pBH01_F1.FOR	AATTTCAGCACTTGCTCCTGCTGGAAG
2	pBH01_F1.REV	TAGACAGATCTATCGATGCATGCCATGGTATCAACATAATCACCCTCTTCCAAATCAATCA
3	pBH01_F2.FOR	TTCTACTGCTAAGTAAAAAATTATTTTATTTTTCTAATAGTAATATAACTATCAATAGGACTATATGG
4	pBH01_F2.REV	GGACTTCCAGCAGGAGCAAGTGCTGAA
5	pBH01_F3.FOR	ATATCGGATCCATATGACGTCGACGCGTCTTTTACTTGATCATTTAGTAAATCATATTTTTTAAAATTCTCTTGTACTTG
6	pBH01_F3.REV	CTATTAGAAAAATAAAATAATTTTTTACTTAGCAGTAGAACTGTTATCAGTTTTACT
7	pBH01_Vector.FOR	AGATGACGACCATCAGGGACAG
8	pBH01_Vector.REV	AGTGTAAAAGAGTTGATAAATGATTATATTGGGAC
9	pBH04_Gen.rev	CAATATAAAGGTTTCCCGTTAGAATCCATCGCAAGAT
10	pBH04_Gen.fwd	CACCTACATATTGGACGAGTTCAGGAGG

**Figure 1.**
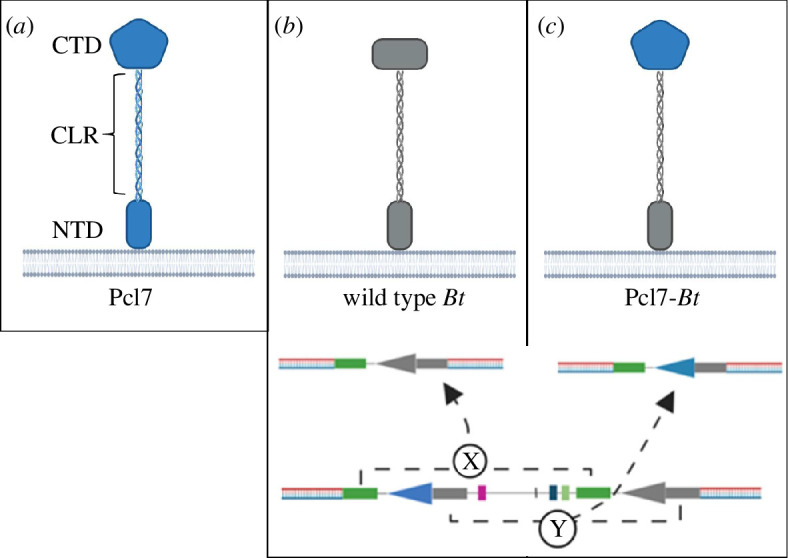
Graphic overview of the construction of the *Pcl7-Bt* fusion. (*a*) *Pcl7* consists of three domains: an amino-terminal domain (NTD), a central collagen-like region (CLR) and a carboxy-terminal globular domain (CTD). (*b*) Homologous recombination can occur either through homologous regions ‘X’, resulting in the regeneration of the wild-type gene and protein (*c*) or through ‘Y’, resulting in the generation of the *Pcl7-Bt* fusion. Figure created with Biorender.com.

The allelic exchange procedure was done as described [53] with some modifications. *B. thuringiensis* integrants were isolated by shifting transformants obtained by electroporation from 28 to 37°C in the presence of 5 µg ml^−1^ erythromycin. pMAD-I-SceI cannot replicate in *B. thuringiensis* at 37 °C, so only cells which integrated the plasmid into the chromosome could propagate. Colonies were screened for the orientation of their insert using PCR (with primers 9 and 10, table 2). Colonies that harboured the right-hand insert were pooled and inoculated in 4 ml fresh TSB medium containing 5 µg ml^−1^ erythromycin at 37°C, 180 r.p.m. overnight. The overnight culture was diluted 1:100 in 50 ml fresh TSB medium containing 5 µg ml^−1^ erythromycin and incubated at 37°C, 180 r.p.m. to an OD_600 nm_ of 0.8 to prepare electrocompetent cells. The cells were transformed with pBKJ223 and plated on TSB agar containing 8 µg ml^−1^ tetracycline. Colonies were pooled and inoculated in 4 ml fresh TSB medium containing 8 µg ml^−1^ tetracycline at 37°C, 180 r.p.m. for 6 h. Serial dilutions (10^−1^, 10^−2^, 10^−3^ and 10^−4^) were prepared and 100 µl of each dilution was plated on TSB agar plates containing 8 µg ml^−1^ tetracycline. Single colonies were patched on TSB agar with 8 µg ml^−1^ tetracycline as well as TSB agar with 5 µg ml^−1^ erythromycin to screen for loss of erythromycin resistance. Erythromycin-sensitive clones were picked and stored in 50 µl LB-Glycerol (15%) at −20°C. The clones were screened for the desired insert (figure 1 *c*; recombination through homologous regions ‘Y’) using primers 9 and 10. Clones with confirmed allelic exchange were inoculated into 4 ml fresh TSB media and incubated at 37°C, 180 r.p.m. overnight. Genomic DNA was prepared and Sanger-sequenced (Microsynth AG, Balgach, Switzerland). Correct clones were inoculated in 4 ml fresh TSB and incubated at 37°C, 180 r.p.m. overnight. The next day freezer stocks were generated and stored at −80°C for later use.

### 2.5. Sporulation and purification of spores

Spores were purified as described by [57] with some modifications. An overnight culture of *B. thuringiensis* in 4 ml of TSB was grown at 37°C, 180 r.p.m. The overnight culture was diluted 1:100 in 50 ml fresh TSB media containing 0.1 mmol MnSO_4_ (PanReac, AppliChem) and grown at 37°C, 180 r.p.m. for 10 days. Cultures were transferred to a 50 ml falcon tube and stored on ice for 20 min. The tubes were spun down at 4°C, 10 000 r.p.m. for 10 min. The pellet was suspended in 20 ml of 50 mM Tris–HCL (pH 7.2; PanReac, AppliChem) with an addition of 50 µg ml^−1^ lysozyme (from hen egg whites, Fluka). Samples were incubated at 37°C, 180 r.p.m. for 1 h. The sample was spun down at 4°C, 10 000 r.p.m. for 10 min, and the pellet was suspended in cold sterile water. This washing step was repeated twice. The pellet was then suspended in 5 ml 0.05% SDS solution (Sigma, D6750-10G) by vortexing and incubated for 5 min at room temperature. The sample was then spun down at 4°C, 10 000 r.p.m. for 10 min, and the pellet was suspended in cold sterile water. This washing step was repeated five times. After the final washing step, the pellet was suspended in 5 ml of cold, sterile water and stored at 4°C for later use. Spores were counted using a Neubauer-improved chamber (Paul Marienfeld GmbH, Lauda-Konigshofen, Germany) with a chamber depth of 0.1 mm.

### 2.6. Fluorescent labelling of spores

*Pasteuria ramosa* spores were isolated by homogenizing infected *Daphnia* in ADaM followed by centrifugation at room temperature, 8000 r.p.m. for 5 min. *Bacillus thuringiensis* spores were thawed and centrifuged at room temperature, 8000 r.p.m. for 5 min. *Pasteuria ramosa* or *B. thuringiensis* pellets were suspended in 0.8 ml of 0.1 M sodium bicarbonate (pH 9.1; Sigma, S5761-500G) with 2 mg ml^−1^ of fluorescein-5(6)-isothiocyanate (Sigma-Aldrich, St Louis, MO). The samples were then incubated in the dark at room temperature, 1600 r.p.m. shaking for 2 h, followed by centrifugation at room temperature, 8000 r.p.m. for 4 min. The pellet was suspended in 0.8 ml sterile water and centrifuged at room temperature, 8000 r.p.m. for 4 min; the supernatant was then removed. This washing step was repeated three times. Spore suspensions were stored in sterile water at 4°C in the dark for further use.

### 2.7. Attachment assay

*Daphnia* were individually placed into a 96-well plate containing 150 µl of ADaM per well. Spore solution (10 µl) containing **~**500 fluorescently labelled *P. ramosa* spores was added to each well and incubated in the dark for 5 min. For *B. thuringiensis*, 10 µl of spore solution containing **~**50 000 labelled *B. thuringiensis* spores was added to each well and incubated in the dark for 5 min. The entire liquid volume in each well was removed and replaced with 150 µl fresh ADaM. This washing step was repeated twice, after which the entire liquid volume in each well was removed. The *Daphnia* were placed individually on a microscopy slide using a toothpick. A glass cover slide was applied to the *Daphnia* gently to avoid crushing it. Spore attachment was assessed by carefully observing the entire depth of the field of vision in transparent animal, while the animal was alive.

To demonstrate the different phenotypes, we took pictures through the transparent body wall of the *Daphnia*. Using living animals is necessary to see the spores in the oesophagus, but puts limits on the quality of the pictures, as animals move and the body tissues surrounding the oesophagus blur the picture. Images were taken using Leica Application Suite (v. 4.12, using package ‘montage’) with a Leica DM6 B (Leica Microsystems, Wetzlar, Germany) microscope fitted with a Leica DFC 7000T camera and a GFP Filter cube (Excitation Filter BP 470/40).

### 2.8. Competitive attachment assay

*Daphnia* were individually placed into a 96-well plate containing 150 µl of ADaM per well. We then added 10 µl of spore solution to each well according to each treatment—for C1/C19: 50 labelled *P. ramosa* spores; for WT-*Bt* + C1/C19: **~**20 000 labelled *B. thuringiensis* spores followed by 50 labelled *P. ramosa* spores; and for *Pcl7-Bt* + C1/C19: **~**20 000 labelled *Pcl7-Bt* spores followed by 50 labelled *P. ramosa* spores—and incubated them in the dark for 5 min. The entire liquid volume in each well was removed and replaced with 150 µl fresh ADaM. This washing step was repeated twice to remove excess spores. The *Daphnia* were then placed individually on a microscopy slide and a glass cover slide was gently applied to the *Daphnia* to avoid crushing them. The number of *P. ramosa* spores attached to the *D. magna* oesophagus were then counted by manually scanning the z-stacks.

### 2.9. Selection of candidate collagen-like proteins

The N-terminal domain and collagen-like region of 3 out of 8 CLPs in *B. thuringiensis* (table 3) were selected based on the presence of the sequence motif ‘LVGPTLPPIPP’, likely required for incorporation into the exosporium, to construct fusions with the *pcl7* CTD.

**Table 3.** Candidate collagen-like proteins in *B. thuringiensis*.

locus tag	product	motif
*BTB_c12600*	*collagen-like exosporium surface protein*	*LVGPTLPPIPP*
BTB_c24780	triple helix repeat-containing collagen	not present
BTB_c34100	collagen triple helix repeat protein	not present
*BTB_c35490*	*collagen triple helix repeat protein*	*LIGPTLPSIPP*
BTB_c37810	collagen triple helix repeat protein	not present
*BTB_c38740*	*triple helix repeat-containing collagen*	***I*** *IGPTLPP* ***V*** *PP*
BTB_c46750	collagen triple helix repeat domain protein	not present
BTB_c48800	collagen triple helix repeat domain protein	not present

*Notes:* Eight candidate *B. thuringiensis* genes with similar N-terminal domains and amino acid sequences as bclA. Deviations from the specific sequence motif, likely required for incorporation into the exosporium, are marked in bold. Genes selected for the construction of fusions with *pcl7* are highlighted in italic. Sequences were obtained from GenBank [58] (accession no. CP003889).

## 3. Results

To validate the role of the CTD of the Pcl7 of *P. ramosa* as an adhesin that recognizes the host *Daphnia magna* in a genotype-specific manner, we employed surface presentation on *B. thuringiensis* spores. Specifically, we fused the Pcl7-CTD to the N-terminal domain of the main collagen-like exosporium surface protein of *B. thuringiensis* (BTB_c12600, table 3). Compared to other potential fusion partners with collagen-like domain (BTB_c35490, BTB_c38740, table 3) BTB_c12600 is more similar in its N-terminal collagen-like domain to Pcl7 and contains a peptide that matches perfectly to the consensus motif for incorporation in the surface exosporium. We identified the CTD of BTB_c12600 in *Bt* 407 as the N-terminal 166 amino acids after the last collagen-like repeat unit (table 4). We replaced this CTD in the chromosome of *Bt* 407 with the 167 amino acid-long CTD of Pcl7 using two consecutive single cross-overs. The construct was verified by sequencing. The resulting recombinant Pcl7-*Bt* strain showed normal growth and sporulation. We induced spore formation and purified spores using standard procedures.

**Table 4.** Amino acid sequences used for the Pcl7-Bt fusion.

protein	amino acid sequence	length
Bt 407 BTB_c12600	*MSNNNYSNGLNPDESLSASAFDPNLVGPTLPPIPPFTLPTGPTGPTGPTGPTGPTVPTGPTGPTGPTGPTGPTGPT* *GPTGPTGPTGDTGTTGPTGPTGDTGATGPTGPTGDTGATGPTGPTGDTGATGPTGPTGDTGATGPTGPTGDTGA* *TGPTGPTGATGPTGPT*GPSGLGLPAGLYAFNSAGISLDLGLNAPVPFNTVGSQFGTAISQLDADTFVIAETGFYKITVIVYTAAISVLGGLTIQVNGVSVPGTGATLISVGAPIVVQAITQITTTPSLVEVIVTGLGLSLALGTNASIIIEKVA*	305 AA
Pcl7	MMSILVGPTGPTGPTGDTGVPGGAIGITGPTGQSMTGIMGNQGPSGIQGPTGITGITGPTGITGITGITGISITGPTGTTGFTGITGPTGVTGPTGETGPVIFISGITGPTGPTGPTGVNITGSTGITGSQGITGNTGLQGPQGPQISPGPSGIQGNQGPIGPT**ASAEIASFRRFTLANVTTFTTPVNFNSQFNLSSSISLLSNNTDISIQPGTYIFNFGGLLYSAGGGGAGANESAVY** LSLVSGSLNTYGTNIKQPYGFASTLTRQNTSASAYGYGGMLYQVAEYMIQVTAAAVIRMLLFNASSYTMTPAQLMLPYSPIDSYITIRKIK*	331 AA
Pcl7-Bt fusion	*MSNNNYSNGLNPDESLSASAFDPNLVGPTLPPIPPFTLPTGPTGPTGPTGPTGPTVPTGPTGPTGPTGPTGPTGP* *TGPTGPTGPTGDTGTTGPTGPTGDTGATGPTGPTGDTGATGPTGPTGDTGATGPTGPTGDTGATGPTGPTGDT* *GATGPTGPTGATGPTGPT* **ASAEIASFRRFTLANVTTFTTPVNFNSQFNLSSSISLLSNNTDISIQPGTYIFNFGGLLY** **SAGGGGAGANESAVYLSLVSGSLNTYGTNIKQPYGFASTLTRQNTSASAYGYGGMLYQVAEYMIQVTAAAVIRML** LFNASSYTMTPAQLMLPYSPIDSYITIRKIK*	333 AA

*Notes:* Amino acid sequences of the native Bt collagen-like exosporium surface protein BTB_c12600, the Pcl7 protein and the resulting Pcl7-Bt fusion protein. The Bt NTD used in the fusion protein is highlighted in italic and the Pcl7 CTD in bold. Asterisk represents the stop codon.

**Figure 2.**
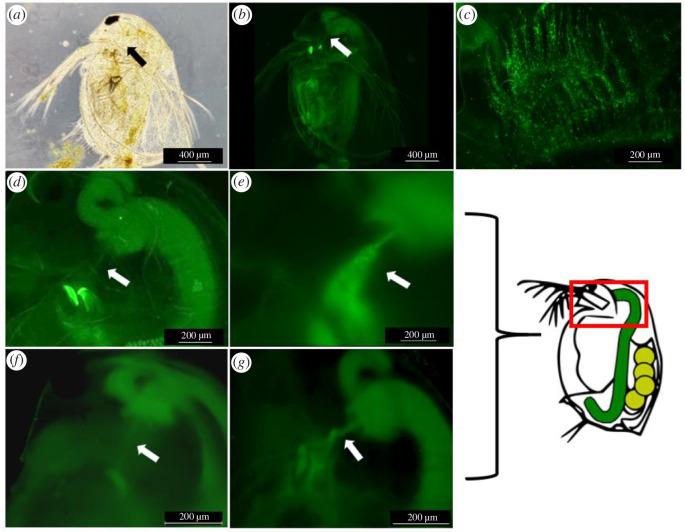
Attachment phenotypes using labelled spores. Microscopy images demonstrating the various attachment phenotypes using labelled spores. The red box in the schematic to the right indicates the approximate area (head region) of images (*d*–*g*). (*a*) Bright-field image of a *D. magna* (genotype (=clone) HU-HO-2) showing the entire animal with an arrow indicating the site of the oesophagus. (*b*) Overview of a *D. magna* (genotype HU-HO-2) using a GFP Filter cube showing the entire animal with an arrow indicating the location of the oesophagus. Note the weak autofluorescence of the *D. magna* tissue. (*c*) Labelled *B. thuringiensis* vegetative cells accumulated in the filter setae of a *D. magna* (genotype HU-HO-2). (*d*) Closeup of the *D. magna* foregut with an arrow indicating the position of the oesophagus, situated perpendicular to the arrow. The two bright objects left of the arrow are the autofluorescent mandibles. (*e*) Upper body of a susceptible *D. magna* (genotype HU-HO-2) with labelled C1 *P. ramosa* spores attached to the oesophagus (arrow). (*f*) Upper body of a resistant *D. magna* (genotype FI-Xinb3) where labelled Pcl7-*Bt* spores do not aggregate in the oesophagus (arrow). The faint visible light fluorescent band is attributed to autofluorescence. The beginning of the midgut, visible in the upper right corner, shows fluorescence because labelled spores have been ingested by the *Daphnia*. (*g*) Upper body of a susceptible *D. magna* (genotype HU-HO-2) with labelled Pcl7-*Bt* spores attached to the oesophagus (arrow). The midgut with its appendix (=caecum) is visible on the right.

**Figure 3.**
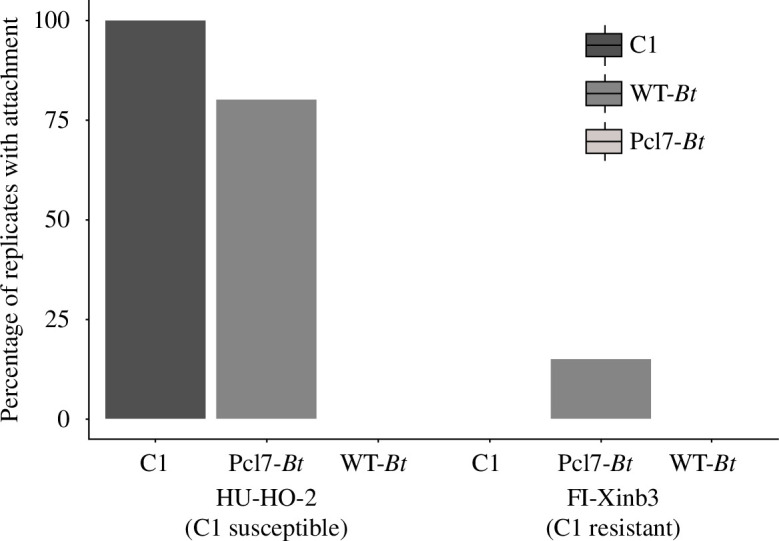
Attachment assay. *D. magna* that are both susceptible (genotype HU-HO-2) and resistant (genotype FI-Xinb3) to C1 *P. ramosa* were exposed to different bacteria: around 5000 labelled C1 *P. ramosa* spores (C1), ~50 000 labelled *B. thuringiensis* WT-*Bt* spores (WT-*Bt*) or ~50 000 labelled Pcl7-*Bt* spores (Pcl7-*Bt*). Each isolate was tested on 20 host individuals for each resistotype (*n* = 20, 120 individuals in total).

To determine adherence of *P. ramosa* and wild-type (WT-*Bt*) or *pcl7*-expressing (Pcl7-*Bt*) *B. thuringiensis* spores to *D. magna*, we covalently labelled the bacteria with fluorescein and tracked them in infection assays with different *D. magna* genotypes using fluorescence microscopy [13]. C1 *P. ramosa* spores attached to the oesophagus of susceptible *D. magna* (figure 2*e*; 100% attachment, figure 3) but not to resistant hosts (figure 3) as previously observed. Vegetative WT-*Bt* and Pcl7-*Bt* cells showed no attachment to the *D. magna* oesophagus but can be observed in the filter setae of both susceptible and resistant *Daphnia* (figure 2*c*), possibly being trapped in the mucus that lines the filter-feeding apparatus. WT-*Bt* spores showed attachment to neither the susceptible nor resistant host (0% attachment, figure 3), while Pcl7-*Bt* spores attached to the oesophagus of susceptible *D. magna* (figure 2*g*). Pcl7-*Bt* attached at low frequency and density to resistant *D. magna* (figure 2*f*; 15% attachment, figure 3), possibly suggesting incorrect glycosylation of Pcl7 [19] in the heterologous *B. thuringiensis* system. Attachment of Pcl7-*Bt* spores to other tissues or known sites of *P. ramosa* attachment such as the hindgut or the external postabdomen [59] was not observed. Thus, a single domain of CLP from *P. ramosa* was sufficient to mediate *Pasteuria*-like adhesion properties for recombinant *B. thuringiensis* spores.

**Figure 4.**
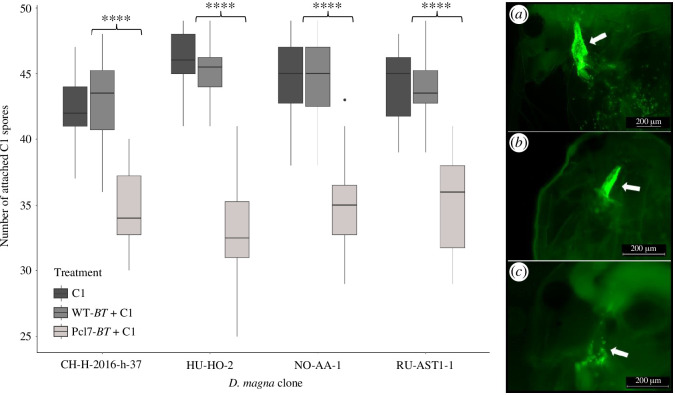
Competitive attachment assay for C1 *P. ramosa*. ‘C1’—individuals were exposed to 50 labelled C1 *P. ramosa* spores (*a*); ‘WT-*Bt* + C1’—individuals were exposed to ~20 000 *B. thuringiensis* WT-*Bt* spores prior to the addition of 50 C1 *P*. *ramosa* spores (*b*); ‘Pcl7*−Bt* + C1’—individuals were exposed to ~20 000 Pcl7-*Bt* spores prior to the addition of 50 C1 *P*. *ramosa* spores (*c*). Each treatment was performed using 20 individual *D. magna,* and the assay was repeated for four different host genotypes (*n* = 20, 60 individuals per genotype (=clone), 240 individuals in total). A *t*‐test was performed to compare the WT-*Bt* + C1 against Pcl7-*Bt* + C1 means. **** *t*‐Test is significant with a *p*‐value of <10^−8^. The boxplots display the 25th percentile, median and 75th percentile, while the whiskers display the minimum (Q1 −1.5*IQR) and maximum (Q3 +1.5*IQR). Outliers are shown as dots. Statistical analysis was done using R (V. 4.1.0, with the R-base package and R packages ‘ggubr’ and ‘ggplot2’).

To determine if Pcl7 presented on *B. thuringiensis* spores can block *P. ramosa* adhesion to *D. magna*, we infected various *D. magna* genotypes (table 1) with 50 spores of two different *P. ramosa* clones after incubation with a 400-fold excess of the much smaller WT-*Bt* or Pcl7-*Bt* spores. After 5 min exposure, we counted the number of *P. ramosa* spores attached to the *D. magna* oesophagus. WT-*Bt* had no impact on subsequent *P. ramosa* adhesion for all tested *D. magna* and *P. ramosa* genotypes (figure 4). However, Pcl7-*Bt* diminished adhesion of C1 *P. ramosa* spores in four different susceptible *D. magna* genotypes, indicating that Pcl7 was sufficient to block adhesion sites in the host for *P. ramosa*.

**Figure 5.**
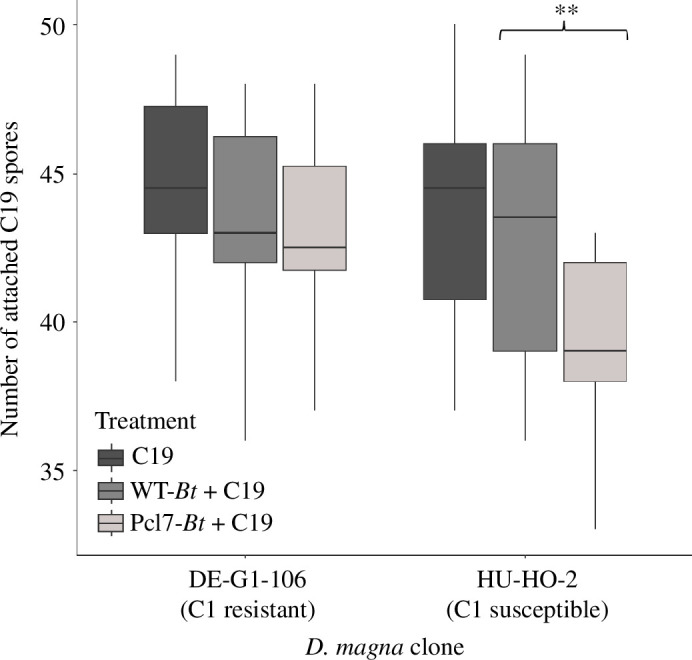
Competitive attachment assay for C19 *P. ramosa*. ‘C19’—idividuals were exposed to 50 labelled C19 *P. ramosa* spores; ‘WT-*Bt* + C19’—individuals were exposed to ~20 000 WT-*Bt* spores prior to the addition of 50 C19 *P*. *ramosa* spores; ‘Pcl7-*Bt* + C19’—individuals were exposed to ~20 000 Pcl7-*Bt* spores prior to the addition of 50 C19 *P*. *ramosa* spores. Each treatment was performed using 20 individual *D. magna,* and the assay was repeated for two different genotypes (*n* = 20, 60 individuals per genotype and 120 individuals in total). ** *t*‐Test, *p* < 0.01.

We also tested Pcl7-*Bt* against C19 *P. ramosa*, an isolate that shows a different host genotype dependence from C1 *P. ramosa* (the source of Pcl7 in Pcl7-*Bt*). Pcl7-*Bt* had no impact on C19 adhesion to *D. magna* DE-G1-106 (susceptible to *P. ramosa* C19; resistant to C1), but diminished C19 adhesion to *D. magna* HU-HO-2 (susceptible to *P. ramosa* C19 and susceptible to C1) (figure 5). Thus, Pcl7-*Bt* could prevent *P. ramosa* adhesion specifically in *D. magna* resistotypes with a Pcl7-receptor (enabling infection by C1), but not in *D. magna* resistotypes without such a receptor. As C19 uses a different receptor from C1, the blocking in HU-HO-2 was probably mediated by steric hindrance between adhering Pcl7-*Bt* and *P. ramosa*.

## 4. Discussion

**Figure 6.**
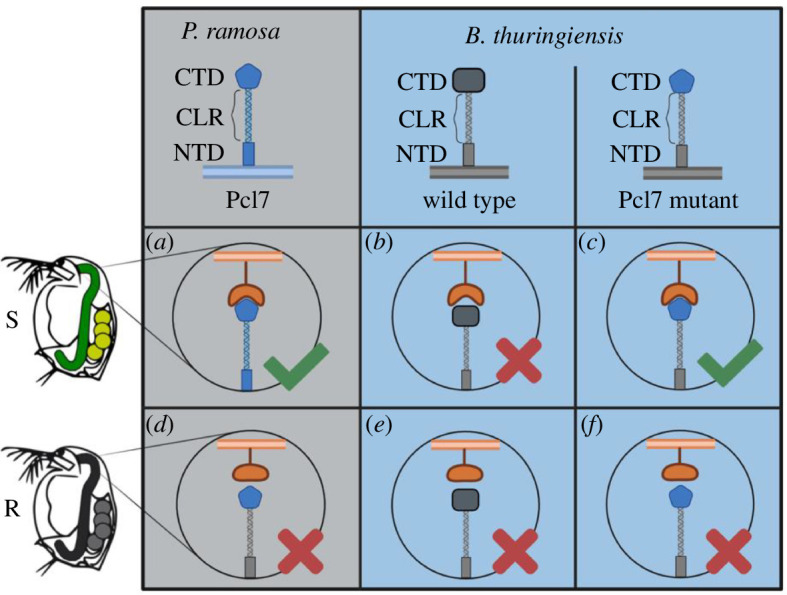
Proposed mechanism for Pcl7-mediated attachment. (*a*) C1 *P. ramosa* ectopically expresses Pcl7 and attaches to the oesophagus of susceptible (S) *D. magna* by binding to host cell surface moieties. (*b*) WT-*Bt* spores do not attach to the oesophagus of susceptible *D. magna* due to the lack of a compatible moiety on the host cell surface. (*c*) Pcl7-*Bt* spores, harbouring the Pcl7 fusion protein attach to the oesophagus of susceptible *D. magna* by binding to the same cell surface moieties as the original Pcl7. (*d*) C1 *P. ramosa* does not attach to the oesophagus of resistant (R) *D. magna*. (*e*) WT-*Bt* spores and (*f*) Pcl7-*Bt* spores do not attach to the oesophagus of resistant *D. magna*. Figure created with Biorender.com.

The mechanism underlying specific coevolution by negative frequency-dependent selection is believed to be driven by the interaction between host and parasite genes. Identifying these genes marks a major step towards understanding this mechanism. In the *Daphnia–Pasteuria* system coevolution is well characterized on a phenotypic level, but the underlying genes are still largely unknown. Here, we tested and confirmed the hypothesis that a CLP is crucial for the attachment of the *Pasteuria* parasite to the cuticle of its *Daphnia* host. Polymorphism in the attachment phenotype had previously been shown to be the most important step in the coevolution of the two antagonists [13,18], and a specific CLP (Pcl7) of *P. ramosa* was linked to the attachment polymorphism [19]. Here, we used *B. thuringiensis* as a surrogate to express a functional fusion protein harbouring the globular domain of Pcl7. By replacing a single C-terminal sequence in *B. thuringiensis* with the one from *Pasteuria* Pcl7, we were able to create *B. thuringiensis* spores capable of attaching *in vivo* to the host’s oesophagus wall. This result is in line with previous studies that have sought to understand CLP function in bacterial infections using deletion mutants for CLPs [60] or purifying recombinant CLPs [35]. Our Pcl7-*Bt* spores not only attached well to four susceptible host genotypes (figures 3 and 4), but they also attached only sparsely to two resistant host genotypes (figures 3 and 5), revealing a very similar specificity to that of *P. ramosa* (figure 6).

Proteins associated with the spore coat in *B. thuringiensis*, including the BclA homologues that were altered in this study, are predominantly synthesized during sporulation [43]. We anticipated that the Pcl7 fusion protein would be present upon completed spore formation. Consistent with this, only spores but not vegetative Pcl7-*Bt* cells adhered to the host’s oesophagus. The specificity of the *B. thuringiensis* spores to a single host tissue thus indicates the functional ectopical expression of the Pcl7 fusion protein, generating the same attachment phenotype as C1 *P. ramosa* spores. This attachment specificity was only seen in hosts susceptible to C1 *P. ramosa*, while in two resistant host genotypes, attachment was either weak or entirely absent. Thus, by replacing part of a single protein, we were able to transform *B. thuringiensis* from non-attaching to attaching, recreating the first step of the infection process. To test if Pcl7-*Bt* spores adhere to the same molecular structure in the oesophagus wall as C1 *P. ramosa* spores we conducted competitive attachment assays. We found that the moieties or potential receptors on the *D. magna* oesophagus cuticle surface are blocked by the Pcl7-*Bt* spores, supporting our hypothesis that the Pcl7-*Bt* interferes with the attachment sites of C1 *P. ramosa*.

The specificity of the Pcl7-*Bt*’s attachment was less pronounced than that of the original *P. ramosa* pathogen, with the difference in attachment between a resistant and a susceptible host genotype being 0% and 100% for C1 *P. ramosa*, while it was 15% and 80% for the Pcl7-*Bt* spores (figure 3). Furthermore, the competitive attachment assays showed that C1 spores were not totally blocked from attachment. Some *Pasteuria* spores were still able to attach after the host had been treated with the Pcl7-*Bt* spores. Finally, to see a visible picture of attachment, we needed much higher spore concentrations of the Pcl7-*Bt*s than the bigger *P. ramosa* pathogen. However, the weak signals observed are certainly caused in part by the rapid germination of *B. thuringiensis* spores once exposed to the *Daphnia*, as germinating spores do not express BclA [43]. Furthermore, because *P. ramosa* spores are large (about 5.5 µm in diameter) and thus much more visible compared to the *B. thuringiensis* spores (about 1.6 × 0.8 µm) [61], more spores are required to see attachment with the fluorescent microscope.

The difference in the attachment of Pcl7-*Bt* spores as compared to spores from C1 *Pasteuria* indicates the presence of factors that contribute to the attachment. The specific coevolution of *P. ramosa* with *D. magna* might have streamlined the system to a very high degree, with Pcl7 certainly playing a vital role, but for a stronger attachment, other factors are needed. One such factor may be glycosylation. BclA, and CLPs in general, are known to be highly glycosylated [62,63]. The sequence polymorphism of Pcl7 results in two predicted N-linked glycosylation sites, one of which correlates with the infection phenotype of *Pasteuria* [19] and thus may partly explain the attachment polymorphism. The Pcl7 fusion protein expressed in *B. thuringiensis* may contain different glycans from Pcl7 expressed in *P. ramosa* or might not be glycosylated at all. This might result in altered adhesion properties. Other fusion proteins containing other sequence polymorphisms or glycosylation sites might be used in the future to work out the details of these differences. An analysis of the specific carbohydrate moieties of the Pcl7 glycoprotein could also be done to support this hypothesis [64]. However, our data show that such a potential mis-glycosylation had only a minor impact on adhesion and competition. Other factors that may contribute to attachment could be related to the spore morphology. *P. ramosa* spores have an unusual shape, which may have evolved to maximize adhesion.

Our study verified that the globular part of the Pcl7 protein contributes decisively to the parasite’s ability to attach to the host cuticle, although the molecular mechanisms for the attachment polymorphism are still unclear, as is the composition of the host cuticle surface in the oesophagus. Previous research has shown that CLPs can bind to a variety of molecules and cell surface components such as integrins [28], glycoproteins [34], polysaccharides [65], lipoproteins [35] and mammalian collagen [66]. Future studies could focus on identifying the *D. magna* receptor for Pcl7.

## Data Availability

All data reported in this article are available online [67].

## References

[1] Jack R , Du Pasquier L . 2019 Evolutionary concepts in immunology. Cham, Switzerland: Springer Nature Switzerland. (10.1007/978-3-030-18667-8)

[2] Poulin R . 2007 Evolutionary ecology of parasites. Princeton, NJ: Princeton University Press. (10.1515/9781400840809)

[3] Schmid-Hempel P . 2021 Evolutionary parasitology, 2nd edn. Oxford, UK: Oxford University Press. (10.1093/oso/9780198832140.001.0001)

[4] Ebert D , Fields PD . 2020 Host–parasite co-evolution and its genomic signature. Nat. Rev. Genet. **21** , 754–768. (10.1038/s41576-020-0269-1) 32860017

[5] Vorburger C , Perlman SJ . 2018 The role of defensive symbionts in host–parasite coevolution. Biol. Rev. Camb. Philos. Soc. **93** , 1747–1764. (10.1111/brv.12417) 29663622

[6] Thrall PH , Barrett LG , Dodds PN , Burdon JJ . 2015 Epidemiological and evolutionary outcomes in gene-for-gene and matching allele models. Front. Plant Sci. **6** , 1084. (10.3389/fpls.2015.01084) 26779200 PMC4703789

[7] Woolhouse MEJ , Webster JP , Domingo E , Charlesworth B , Levin BR . 2002 Biological and biomedical implications of the co-evolution of pathogens and their hosts. Nat. Genet. **32** , 569–577. (10.1038/ng1202-569) 12457190

[8] Ebert D . 2018 Open questions: what are the genes underlying antagonistic coevolution? BMC Biol. **16** , 114. (10.1186/s12915-018-0583-7) 30382846 PMC6211428

[9] Ameline C . 2020 Evolution and genetic architecture of resistance in a natural population of Daphnia magna undergoing strong epidemics of the bacteria Pasteuria ramosa. PhD thesis, University of Basel, Switzerland.

[10] Auld SKJR , Tinkler SK , Tinsley MC . 2016 Sex as a strategy against rapidly evolving parasites. Proc. R. Soc. B **283** , 20162226. (10.1098/rspb.2016.2226) PMC5204169 28003455

[11] Decaestecker E , Gaba S , Raeymaekers JAM , Stoks R , Van Kerckhoven L , Ebert D , De Meester L . 2007 Host-parasite ‘Red Queen’ dynamics archived in pond sediment. Nature **450** , 870–873. (10.1038/nature06291) 18004303

[12] Bento G , Routtu J , Fields PD , Bourgeois Y , Du Pasquier L , Ebert D . 2017 The genetic basis of resistance and matching-allele interactions of a host–parasite system: the Daphnia magna–Pasteuria ramosa model. PLoS Genet. **13** , e1006596. (10.1371/journal.pgen.1006596) 28222092 PMC5340410

[13] Duneau D , Luijckx P , Ben-Ami F , Laforsch C , Ebert D . 2011 Resolving the infection process reveals striking differences in the contribution of environment, genetics and phylogeny to host–parasite interactions. BMC Biol. **9** , 11. (10.1186/1741-7007-9-11) 21342515 PMC3052238

[14] Luijckx P , Fienberg H , Duneau D , Ebert D . 2013 A matching-allele model explains host resistance to parasites. Curr. Biol. **23** , 1085–1088. (10.1016/j.cub.2013.04.064) 23707426

[15] Bento G , Fields PD , Duneau D , Ebert D . 2020 An alternative route of bacterial infection associated with a novel resistance locus in the Daphnia-Pasteuria host-parasite system. Heredity **125** , 173–183. (10.1038/s41437-020-0332-x) 32561843 PMC7490384

[16] Fredericksen M , Fields PD , Du Pasquier L , Ricci V , Ebert D . 2023 QTL study reveals candidate genes underlying host resistance in a Red Queen model system. PLoS Genet. **19** , e1010570. (10.1371/journal.pgen.1010570) 36730161 PMC9894429

[17] McElroy K , Mouton L , Du Pasquier L , Qi W , Ebert D . 2011 Characterisation of a large family of polymorphic collagen-like proteins in the endospore-forming bacterium Pasteuria ramosa. Res. Microbiol. **162** , 701–714. (10.1016/j.resmic.2011.06.009) 21726633

[18] Ebert D , Duneau D , Hall MD , Luijckx P , Andras JP , Pasquier L , Ben-Ami F . 2016 A population biology perspective on the stepwise infection process of the bacterial pathogen Pasteuria ramosa in Daphnia. Adv. Parasitol. **91** , 265–310. (10.1016/bs.apar.2015.10.001) 27015951

[19] Andras JP , Fields PD , Du Pasquier L , Fredericksen M , Ebert D . 2020 Genome-wide association analysis identifies a genetic basis of infectivity in a model bacterial pathogen. Mol. Biol. Evol. **37** , 3439–3452. (10.1093/molbev/msaa173) 32658956 PMC7743900

[20] Mouton L , Traunecker E , McElroy K , Du Pasquier L , Ebert D . 2009 Identification of a polymorphic collagen-like protein in the crustacean bacteria Pasteuria ramosa. Res. Microbiol. **160** , 792–799. (10.1016/j.resmic.2009.08.016) 19770039

[21] Qiu Y , Zhai C , Chen L , Liu X , Yeo J . 2023 Current insights on the diverse structures and functions in bacterial collagen-like proteins. ACS Biomater. Sci. Eng. **9** , 3778–3795. (10.1021/acsbiomaterials.1c00018) 33871954

[22] Xu C , Yu Z , Inouye M , Brodsky B , Mirochnitchenko O . 2010 Expanding the family of collagen proteins: recombinant bacterial collagens of varying composition form triple-helices of similar stability. Biomacromolecules **11** , 348–356. (10.1021/bm900894b) 20025291 PMC2818787

[23] Sylvestre P , Couture-Tosi E , Mock M . 2002 A collagen-like surface glycoprotein is a structural component of the Bacillus anthracis exosporium. Mol. Microbiol. **45** , 169–178. (10.1046/j.1365-2958.2000.03000.x) 12100557

[24] Vandersmissen L , De Buck E , Saels V , Coil DA , Anné J . 2010 A Legionella pneumophila collagen-like protein encoded by a gene with a variable number of tandem repeats is involved in the adherence and invasion of host cells. FEMS Microbiol. Lett. **306** , 168–176. (10.1111/j.1574-6968.2010.01951.x) 20370832

[25] Lukomski S , Bachert BA , Squeglia F , Berisio R . 2017 Collagen-like proteins of pathogenic streptococci: streptococcal collagen-like proteins. Mol. Microbiol. **103** , 919–930. (10.1111/mmi.13604) 27997716 PMC5344740

[26] Rasmussen M , Jacobsson M , Björck L . 2003 Genome-based identification and analysis of collagen-related structural motifs in bacterial and viral proteins. J. Biol. Chem. **278** , 32313–32316. (10.1074/jbc.M304709200) 12788919

[27] Thompson BM , Stewart GC . 2008 Targeting of the BclA and BclB proteins to the Bacillus anthracis spore surface. Mol. Microbiol. **70** , 421–434. (10.1111/j.1365-2958.2008.06420.x) 18761690

[28] Humtsoe JO , Kim JK , Xu Y , Keene DR , Höök M , Lukomski S , Wary KK . 2005 A streptococcal collagen-like protein interacts with the α_2_β_1_ integrin and induces intracellular signaling. J. Biol. Chem. **280** , 13848–13857. (10.1074/jbc.M410605200) 15647274

[29] Kailas L et al . 2011 Surface architecture of endospores of the Bacillus cereus/anthracis/thuringiensis family at the subnanometer scale. Proc. Natl. Acad. Sci. USA **108** , 16014–16019. (10.1073/pnas.1109419108) 21896762 PMC3179049

[30] Sylvestre P , Couture-Tosi E , Mock M . 2003 Polymorphism in the collagen-like region of the Bacillus anthracis BclA protein leads to variation in exosporium filament length. J. Bacteriol. **185** , 1555–1563. (10.1128/JB.185.5.1555-1563.2003) 12591872 PMC148075

[31] Bozue J , Moody KL , Cote CK , Stiles BG , Friedlander AM , Welkos SL , Hale ML . 2007 Bacillus anthracis spores of the bclA mutant exhibit increased adherence to epithelial cells, fibroblasts, and endothelial cells but not to macrophages. Infect. Immun. **75** , 4498–4505. (10.1128/IAI.00434-07) 17606596 PMC1951178

[32] Brahmbhatt TN , Janes BK , Stibitz ES , Darnell SC , Sanz P , Rasmussen SB , O’Brien AD . 2007 Bacillus anthracis exosporium protein BclA affects spore germination, interaction with extracellular matrix proteins, and hydrophobicity. Infect. Immun. **75** , 5233–5239. (10.1128/IAI.00660-07) 17709408 PMC2168272

[33] Wang Y , Jenkins SA , Gu C , Shree A , Martinez-Moczygemba M , Herold J , Botto M , Wetsel RA , Xu Y . 2016 Correction: Bacillus anthracis spore surface protein BcIA mediates complement factor H binding to spores and promotes spore persistence. PLoS Pathog. **12** , e1005968. (10.1371/journal.ppat.1005968) 27736971 PMC5063423

[34] Caswell CC , Han R , Hovis KM , Ciborowski P , Keene DR , Marconi RT , Lukomski S . 2008 The Scl1 protein of M6-type group A Streptococcus binds the human complement regulatory protein, factor H, and inhibits the alternative pathway of complement. Mol. Microbiol. **67** , 584–596. (10.1111/j.1365-2958.2007.06067.x) 18093091

[35] Han R , Caswell CC , Lukomska E , Keene DR , Pawlowski M , Bujnicki JM , Kim JK , Lukomski S . 2006 Binding of the low-density lipoprotein by streptococcal collagen-like protein Scl1 of Streptococcus pyogenes. Mol. Microbiol. **61** , 351–367. (10.1111/j.1365-2958.2006.05237.x) 16856940

[36] Karlström A , Jacobsson K , Flock M , Flock JI , Guss B . 2004 Identification of a novel collagen-like protein, SclC, in Streptococcus equi using signal sequence phage display. Vet. Microbiol. **104** , 179–188. (10.1016/j.vetmic.2004.09.014) 15564026

[37] McElroy K , Mouton L , Du Pasquier L , Qi W , Ebert D . 2011 Characterisation of a large family of polymorphic collagen-like proteins in the endospore-forming bacterium Pasteuria ramosa. Res. Microbiol. **162** , 701–714. (10.1016/j.resmic.2011.06.009) 21726633

[38] Stewart GC . 2015 The exosporium layer of bacterial spores: a connection to the environment and the infected host. Microbiol. Mol. Biol. Rev. **79** , 437–457. (10.1128/MMBR.00050-15) 26512126 PMC4651027

[39] Sylvestre P , Couture-Tosi E , Mock M . 2002 A collagen-like surface glycoprotein is a structural component of the Bacillus anthracis exosporium. Mol. Microbiol. **45** , 169–178. (10.1046/j.1365-2958.2000.03000.x) 12100557

[40] Boydston JA , Chen P , Steichen CT , Turnbough CL . 2005 Orientation within the exosporium and structural stability of the collagen-like glycoprotein BclA of Bacillus anthracis. J. Bacteriol. **187** , 5310–5317. (10.1128/JB.187.15.5310-5317.2005) 16030225 PMC1196033

[41] Gohar M , Gilois N , Graveline R , Garreau C , Sanchis V , Lereclus D . 2005 A comparative study of Bacillus cereus, Bacillus thuringiensis and Bacillus anthracis extracellular proteomes. Proteomics **5** , 3696–3711. (10.1002/pmic.200401225) 16167365

[42] Waller LN , Stump MJ , Fox KF , Harley WM , Fox A , Stewart GC , Shahgholi M . 2005 Identification of a second collagen-like glycoprotein produced by Bacillus anthracis and demonstration of associated spore-specific sugars. J. Bacteriol. **187** , 4592–4597. (10.1128/JB.187.13.4592-4597.2005) 15968070 PMC1151769

[43] Peng Q , Kao G , Qu N , Zhang J , Li J , Song F . 2016 The regulation of exosporium-related genes in Bacillus thuringiensis. Sci. Rep. **6** , 19005. (10.1038/srep19005) 26805020 PMC4750369

[44] Srivastava A , Mohan S , Davies KG . 2022 Exploring Bacillus thuringiensis as a model for endospore adhesion and its potential to investigate adhesins in Pasteuria penetrans. J. Appl. Microbiol. **132** , 4371–4387. (10.1111/jam.15522) 35286009 PMC9311801

[45] Giorno R *et al* . 2007 Morphogenesis of the Bacillus anthracis spore. J. Bacteriol. **189** , 691–705. (10.1128/JB.00921-06) 17114257 PMC1797280

[46] Thompson BM , Hsieh HY , Spreng KA , Stewart GC . 2011 The co-dependence of BxpB/ExsFA and BclA for proper incorporation into the exosporium of Bacillus anthracis. Mol. Microbiol. **79** , 799–813. (10.1111/j.1365-2958.2010.07488.x) 21255119 PMC3044595

[47] Tan L , Turnbough CL . 2010 Sequence motifs and proteolytic cleavage of the collagen-like glycoprotein BclA required for its attachment to the exosporium of Bacillus anthracis. J. Bacteriol. **192** , 1259–1268. (10.1128/JB.01003-09) 20038593 PMC2820836

[48] Bao S *et al* . 2015 Construction of a cell-surface display system based on the N-terminal domain of ice nucleation protein and its application in identification of mycoplasma adhesion proteins. J. Appl. Microbiol. **119** , 236–244. (10.1111/jam.12824) 25857598

[49] Jung HC , Lebeault JM , Pan JG . 1998 Surface display of Zymomonas mobilis levansucrase by using the ice-nucleation protein of Pseudomonas syringae. Nat. Biotechnol. **16** , 576–580. (10.1038/nbt0698-576) 9624691

[50] Park TJ , Heo NS , Yim SS , Park JH , Jeong KJ , Lee SY . 2013 Surface display of recombinant proteins on Escherichia coli by BclA exosporium of Bacillus anthracis. Microb. Cell. Fact. **12** , 81. (10.1186/1475-2859-12-81) 24053632 PMC3850424

[51] Sheppard AE , Poehlein A , Rosenstiel P , Liesegang H , Schulenburg H . 2013 Complete genome sequence of Bacillus thuringiensis strain 407 cry-. Genome Announc. **1** , 1. (10.1128/genomeA.00158-12) PMC3569317 23405326

[52] Monk IR , Shah IM , Xu M , Tan MW , Foster TJ . 2012 Transforming the untransformable: application of direct transformation to manipulate genetically Staphylococcus aureus and Staphylococcus epidermidis. mBio **3** , e00277-11. (10.1128/mBio.00277-11) 22434850 PMC3312211

[53] Janes BK , Stibitz S . 2006 Routine markerless gene replacement in Bacillus anthracis. Infect. Immun. **74** , 1949–1953. (10.1128/IAI.74.3.1949-1953.2006) 16495572 PMC1418658

[54] Arnaud M , Chastanet A , Débarbouillé M . 2004 New vector for efficient allelic replacement in naturally nontransformable, low-GC-content, Gram-positive bacteria. Appl. Environ. Microbiol. **70** , 6887–6891. (10.1128/AEM.70.11.6887-6891.2004) 15528558 PMC525206

[55] Ebert D , Zschokke-Rohringer CD , Carius HJ . 1998 Within- and between-population variation for resistance of Daphnia magna to the bacterial endoparasite Pasteuria ramosa. Proc. R. Soc. Lond. B **265** , 2127–2134. (10.1098/rspb.1998.0549)

[56] Gibson DG , Young L , Chuang RY , Venter JC , Hutchison CA , Smith HO . 2009 Enzymatic assembly of DNA molecules up to several hundred kilobases. Nat. Methods **6** , 343–345. (10.1038/nmeth.1318) 19363495

[57] Tavares MB , Souza RD , Luiz WB , Cavalcante RCM , Casaroli C , Martins EG , Ferreira RCC , Ferreira LCS . 2013 Bacillus subtilis endospores at high purity and recovery yields: optimization of growth conditions and purification method. Curr. Microbiol. **66** , 279–285. (10.1007/s00284-012-0269-2) 23183956

[58] Sheppard AE , Poehlein A , Rosenstiel P , Liesegang H , Schulenburg H . 2013 Complete genome sequence of Bacillus thuringiensis strain 407 Cry-. Genome Announc. **1** , e00158-12. (10.1128/genomeA.00158-12) PMC3569317 23405326

[59] Fredericksen M , Ameline C , Krebs M , Hüssy B , Fields PD , Andras JP , Ebert D . 2021 Infection phenotypes of a coevolving parasite are highly diverse, structured, and specific. Evolution **75** , 2540–2554. (10.1111/evo.14323) 34431523 PMC9290032

[60] Zhao X et al . 2015 Collagen-like proteins (ClpA, ClpB, ClpC, and ClpD) are required for biofilm formation and adhesion to plant roots by Bacillus amyloliquefaciens FZB42. PLoS ONE **10** , e0117414. (10.1371/journal.pone.0117414) 25658640 PMC4319854

[61] Carrera M , Zandomeni RO , Fitzgibbon J , Sagripanti JL . 2007 Difference between the spore sizes of Bacillus anthracis and other Bacillus species. J. Appl. Microbiol. **102** , 303–312. (10.1111/j.1365-2672.2006.03111.x) 17241334

[62] Maes E , Krzewinski F , Garenaux E , Lequette Y , Coddeville B , Trivelli X , Ronse A , Faille C , Guerardel Y . 2016 Glycosylation of BclA glycoprotein from Bacillus cereus and Bacillus anthracis exosporium is domain-specific. J. Biol. Chem. **291** , 9666–9677. (10.1074/jbc.M116.718171) 26921321 PMC4850304

[63] Waller LN , Stump MJ , Fox KF , Harley WM , Fox A , Stewart GC , Shahgholi M . 2005 Identification of a second collagen-like glycoprotein produced by Bacillus anthracis and demonstration of associated spore-specific sugars. J. Bacteriol. **187** , 4592–4597. (10.1128/JB.187.13.4592-4597.2005) 15968070 PMC1151769

[64] Geyer H , Geyer R . 2006 Strategies for analysis of glycoprotein glycosylation. Biochim. Biophys. Acta **1764** , 1853–1869. (10.1016/j.bbapap.2006.10.007) 17134948

[65] Duncan C , Prashar A , So J , Tang P , Low DE , Terebiznik M , Guyard C . 2011 Lcl of Legionella pneumophila is an immunogenic GAG binding adhesin that promotes interactions with lung epithelial cells and plays a crucial role in biofilm formation. Infect. Immun. **79** , 2168–2181. (10.1128/IAI.01304-10) 21422183 PMC3125840

[66] Ellison AJ , Dempwolff F , Kearns DB , Raines RT . 2020 Role for cell-surface collagen of Streptococcus pyogenes in infections. ACS Infect. Dis. **6** , 1836–1843. (10.1021/acsinfecdis.0c00073) 32413256 PMC7354224

[67] Ebert D , Huessy B , Bumann D . 2024 Data from: Ectopical expression of bacterial collagen-like protein supports its role as adhesin in host-parasite coevolution. Dryad Digital Repository. (10.5061/dryad.bk3j9kdjk) PMC10987987 38577215

